# The Effect of a Personalized Exercise Program on Muscle Functional Capacity and Quality of Daily Life: A Randomized Pilot Study

**DOI:** 10.3390/ijerph22091344

**Published:** 2025-08-28

**Authors:** Semra Ercan, Türkü Yalçınol, Özge Öngel

**Affiliations:** Department of Physical Therapy and Rehabilitation, Yeditepe University Bagdat Caddesi Polyclinic, Istanbul 34728, Turkey; turku.yalcinol@yeditepe.edu.tr (T.Y.); ozge.ongel@yeditepe.edu.tr (Ö.Ö.)

**Keywords:** muscle functional capacity, personalized exercise programs, safe exercise in older adults, progressive resistance training, psychological stress, overuse

## Abstract

Objectives: This study aimed to examine the effects of a personalized exercise program on muscle functional capacity and daily life activities among individuals of various age groups and health conditions. Methods: A total of 169 participants aged between 16 and 94 years with varying health statuses were enrolled. The EN-Dynamic system—comprising progressive resistive exercise equipment and Enraf Nonius’ Entrack software—was utilized alongside a newly developed exercise programming software. Maximum functional capacity was measured across 13 distinct muscle groups for each participant. Four different questionnaires were administered pre-intervention to evaluate daily activity levels and disease severity. Based on the collected data, a personalized exercise program was generated using a mathematical formula tailored to each muscle group. The program was applied three times per week for 12 weeks (36 total sessions). Following the intervention, the same measurements and questionnaires were repeated and analyzed statistically. Results: Data were analyzed using *t*-test, one-way ANOVA, Pearson correlation analysis, and Bonferroni post hoc tests. A statistically significant improvement in muscle functional capacity was found (t(168) = −30.65, *p* < 0.01; 95% CI: [16.95, 19.31]; Cohen’s d = 2.35, very large effect size). The questionnaire results also demonstrated substantial reductions (e.g., t(168) = 18.51, *p* < 0.01; Cohen’s d = 1.42). Conclusions: These findings suggest that personalized exercise programs can significantly enhance muscle functional capacity while reducing psychological and physical stress and disease severity. Nonetheless, further controlled and longer-term clinical trials comparing traditional and personalized methods are required to confirm these benefits at the population level.

## 1. Introduction

By the year 2030, the population aged 65 and older in the United States is expected to exceed 70 million, with adults over the age of 85 projected to be the fastest-growing age group [1]. Muscle mass loss typically begins around the age of 60, reaching approximately 39% at this age and up to 50% by age 80 [2]. According to Miller et al. (2000), while only 4% of the U.S. population was aged 65 and older in the early 1900s, this figure has now approached 20%. Moreover, approximately 40% of individuals over the age of 65 experience difficulties performing basic activities of daily living [3].

This age-related physical decline underscores the importance of sarcopenia—a condition characterized by the progressive loss of skeletal muscle mass associated with aging [4]. Research conducted by Evans and Campbell [4] indicates that resistance training may not only prevent but also reverse sarcopenia. Several measurement tools have been developed to evaluate sarcopenia, and these assessments have proven useful in determining the effectiveness of exercise programs and treatment protocols [4,5,6].

However, while general assessments of muscle strength and functional capacity are beneficial, they often fall short in evaluating the performance of individual muscle groups. This limitation makes it difficult to fully understand a person’s ability to withstand the physical and psychological stressors of daily life. The lack of targeted measurements for each muscle group also complicates the development of safe and personalized exercise prescriptions [7]. To address this gap, the present study proposes a novel approach based on the concept of muscle functional capacity for the evaluation and enhancement of muscular performance. This approach emphasizes the independent measurement of different muscle groups.

Beyond physical decline, psychological stress has also been shown to contribute to muscle aging. For instance, Puterman et al. [8] reported a link between chronic psychological stress and telomere shortening, a biomarker of accelerated cellular aging. Similarly, Mannion et al. [9] found that psychological distress negatively affected back muscle endurance in patients with chronic low back pain. Although these findings are not yet conclusive, they highlight the need for a more detailed investigation into the relationship between psychological stress and musculoskeletal deterioration.

Other studies suggest that physical activity may offer protective benefits against both the physiological and psychological effects of aging. For example, a comparative study found that both young adults (aged 20–30) and older adults (aged 60–70) experienced comparable improvements in aerobic capacity following a 12-week exercise program [10,11]. These findings indicate that meaningful physical performance gains are possible at any age.

Given that today’s young adults will become the older adults of the future, early preventive interventions are critically important. At the same time, it is essential for current older adults to continue exercising to reduce or manage their chronic disease burden. However, due to the wide variation in age, health status, baseline muscle strength, and daily activity levels, a one-size-fits-all exercise approach is unlikely to be effective [8].

Designing safe and effective exercise protocols for older adults presents numerous challenges, including age-related physiological changes, comorbid conditions, and an elevated risk of injury. Additionally, there is no clear consensus on the optimal frequency, intensity, and structure of exercise for this population [8,10,12]. These challenges point to the need for personalized solutions that consider not only physical capacity but also psychological and environmental barriers to activity. Moreover, the impact of physical and psychological stress on muscle functional capacity has not been sufficiently studied. This study hypothesizes that the accumulation of daily physical and psychological stress may contribute to muscle fatigue, weakness, and ultimately, joint and tendon degeneration. Aging amplifies these effects, often leading to a vicious cycle of pain, limited mobility, and withdrawal from physical activity [13].

As emphasized by Von Korff, avoiding long-term joint damage due to overuse or injury is essential for maintaining a pain-free lifestyle [14]. Therefore, the exercise model proposed in this study aims to enhance individuals’ resilience to both physical and psychological stressors—even among those with deformities or degenerative conditions.

In a study by Sperling, healthy older adults were compared to young adults aged 20–30, and no significant differences were found in grip strength and transverse volar grip endurance between the two groups at age 70 [15]. This finding underscores the indispensable role of exercise in both maintaining health in older adults and reducing disease severity in those with chronic conditions. While healthy older adults can generally continue traditional exercise methods as long as they do not sustain injuries, those with declining health often struggle with conventional physical activities. Therefore, the development of innovative, individualized exercise strategies for this group is of paramount importance.

The exercise method developed for this study was designed with the following key features:

- Based on objective measurements;
- Minimizes practitioner-dependent variability;
- Enables inter-individual comparisons;
- Applicable across all age groups;
- Safe for individuals with comorbid conditions.

The primary aim of this study is to evaluate whether a personalized exercise intervention based on the concept of muscle functional capacity can produce measurable improvements in both objective muscle performance and subjective quality of life across different age groups. The intervention consists of a 12-week program comprising three exercise sessions per week, totaling 36 sessions.

## 2. Materials and Methods

### 2.1. Study Design

This clinical study was designed to evaluate the effectiveness of increasing muscle functional capacity across a diverse sample of individuals representing various demographic and health-related subgroups.

A total of 169 individuals, aged between 16 and 94 years, participated in the study. These participants presented with a wide range of health conditions.

The inclusion criteria covered the following categories:

- Individuals who had previously attempted conventional exercise methods but had discontinued due to injury or lack of effectiveness;
- Older adults who refused to perform even simple physical activities, including walking, out of fear of injury;
- Individuals with restrictions in daily life activities due to existing knee, hip, or lower back conditions;
- Participants who reported rapid fatigue, increased back pain from routine activities, and general difficulty with movement;
- Adults over the age of 65 whose daily functional capacity had significantly declined, making it difficult for them to perform tasks they previously managed with ease;
- Healthy individuals without diagnosed joint degeneration, but who experienced quick fatigue during routine daily activities;
- Individuals advised by healthcare professionals to increase physical activity due to heart disease, diabetes, or obesity, but who were unable to follow these recommendations because of exacerbated joint-related complaints.

The exclusion criteria were the following:

- Individuals undergoing active cancer treatment;
- Those diagnosed with heart failure;
- Individuals who were unable to move independently due to neurological disorders.

All 169 participants applied to the Yeditepe University Bagdat Street Polyclinic for clinical concerns aligning with the inclusion criteria and voluntarily consented to take part in the study. This selection process was designed to reflect a representative sample of the broader population. Informed consent was obtained from all participants in accordance with ethical guidelines, and voluntary participation was emphasized. No participant withdrew due to medical deterioration or social reasons. All participants completed the 12-week program, attending 36 sessions, three times per week.

- No concurrent medical treatment was administered;
- Participants refrained from any form of sports or physical activity, including recreational walking;
- No physically demanding activities, such as gardening, were permitted.

These controls were rigorously maintained to ensure that any observed improvements could be confidently attributed to the implemented exercise program. To assess the long-term sustainability of the intervention’s effects, participants were also instructed to avoid other forms of exercise for a full year following the program. A follow-up evaluation is planned, and the results of this secondary analysis will be presented in a separate publication.

### 2.2. Data Analysis Approach

The collected data were analyzed using SPSS 22.0 (Statistical Package for the Social Sciences) for Windows. Statistical methods applied in the analysis included *t*-tests, one-way analysis of variance (ANOVA), Pearson correlation analysis, and Bonferroni multiple comparison tests. A predetermined significance level of 0.05 was adopted to determine statistical significance. A post hoc power analysis indicated that the sample size (n = 169) was sufficient to detect a large effect size (d > 0.8) with 95% power at α = 0.05.

### 2.3. The Concept of Muscle Functional Capacity

In this study, the concept of muscle functional capacity was introduced to provide a more comprehensive perspective on muscle strength and endurance. This concept encompasses not only physical stamina but also the ability to tolerate the physical and psychological stress encountered in daily life. It is based on the understanding that the strength needed to prevent overuse injuries is influenced not only by physical endurance but also by mental resilience.

Rather than using traditional measures such as one-repetition maximum (1RM) or grip strength—which primarily assess maximal force output—this study emphasized muscle functional capacity, as these conventional measures do not fully capture the demands and stressors of everyday life. Muscle functional capacity is defined here as a muscle’s ability to meet and sustain the demands of daily living, especially when simultaneously exposed to physical and psychological stressors. The aim is to offer a more relevant and practical assessment of an individual’s ability to maintain an active, pain-free lifestyle despite existing health conditions. Although standard functional capacity tests used in the current scientific literature provide some insight, they often fail to objectively evaluate each muscle group independently. Thus, a new approach was needed—one that minimizes dependence on the skill of the practitioner, allows for standardized measurements in daily use, and yields reproducible results. To address these limitations and ensure clinical applicability, we introduced a classification framework based on ideal functional thresholds tailored to real-life stress scenarios. This study was designed as a preliminary pilot investigation to support the development and implementation of such a model in clinical settings. As part of the exercise method, we categorized muscle functional capacity according to percentages of an ideal activity level. To help prevent joint and muscle overuse in daily life, three distinct levels of daily functional activity were defined.

#### 2.3.1. Basic Daily Activity Level (Questionnaire Score Below 40% of the Total Possible Score and Muscle Functional Capacity Between 40% and 60 of the Ideal)

This activity level is appropriate for individuals who do not experience concurrent physical and psychological stress within a given time frame (e.g., a single day or week). It reflects the ability to perform essential, simple daily activities without significant injury risk. If their muscle functional capacity falls below 40% of the ideal, individuals may begin to experience severe fatigue and pain during daily activities, often leading to withdrawal from an active lifestyle.

#### 2.3.2. Moderate Daily Activity Level (Questionnaire Score Below 60% of the Total Possible Score and Muscle Functional Capacity Between 60% and 80 of the Ideal)

This level is suitable for individuals who occasionally encounter physical and psychological stress simultaneously. It represents the capacity to manage daily tasks at a moderate level without significant risk of injury.

#### 2.3.3. High-Intensity Daily Activity Level (Questionnaire Score Above 60% of the Total Possible Score and Muscle Functional Capacity of 80% or More)

This category applies to individuals who regularly experience prolonged physical and mental stress. It indicates the capacity to manage heavy daily activities without substantial risk of injury.

Building upon this classification system, we developed a targeted implementation strategy tailored to the physiological and psychological needs of different populations.

### 2.4. Targeted Implementation Strategy

The primary objective—particularly for older adults who may be unable to engage in traditional exercise programs or sports due to health concerns—is to help them effectively manage moderate levels of stress. This corresponds to achieving 60–80% of the ideal muscle functional capacity. This classification system enables the creation of tailored exercise programs aligned with the unique needs of individuals across various age groups, especially older adults. By applying this structure, the study aims to support a personalized and effective approach to maintaining muscle health and enhancing overall well-being.

### 2.5. Questionnaires

To determine the required level of muscle functional capacity and to assess physical and psychological stress experienced in daily life, four customized questionnaires were administered. These instruments were adapted from previously validated scales and modified for the specific aims of this study.

#### 2.5.1. Modified Psychological Stress Questionnaire (PSM-9)

Adapted from the 9-item scale developed by Lemyre and Tessier [16], this tool evaluates levels of psychological stress. Each item offers 8 response options, with lower scores indicating minimal stress and higher scores indicating severe psychological distress (see Supplementary Material S1).

#### 2.5.2. Quality of Life in Daily Activities Questionnaire

Based on a modified version of the WHOQOL-100 [17], this 28-item instrument measures quality of life in relation to daily functional activities. Each item includes 5 response options. Lower scores reflect fewer impairments, while higher scores indicate more significant difficulty (see Supplementary Material S2).

#### 2.5.3. Physical Stress Questionnaire

Developed by the principal investigator, this 25-item scale assesses short-term activity tolerance and physical demand across different age groups. Each question has five response options. Lower scores indicate less stress, while higher scores indicate greater stress (see Supplementary Material S3).

#### 2.5.4. Disease Severity Questionnaire

Additionally, developed by the principal investigator, this 32-item tool evaluates the severity of musculoskeletal and systemic diseases. All items offer 5 response options, with higher scores reflecting a greater disease burden (see Supplementary Material S4). Together, these four instruments provided a multidimensional profile of each participant’s physical and psychological stress levels, perceived quality of life, and disease burden—crucial variables for tailoring a personalized exercise intervention. The resulting total scores were integrated into a mathematical algorithm used to personalize exercise prescriptions and to match them with objective muscle performance measurements. To reduce response bias, post-intervention questionnaires were administered in a blinded manner. Neither the participant nor the evaluator had access to the baseline (pre-exercise) responses. All responses were securely stored within the software used for personalized planning. Only the total scores were retrieved for statistical analysis, ensuring the objectivity and reliability of the outcome evaluations.

### 2.6. Exercise Program Methodology

Initially, we aimed to define the level of muscle functional capacity that each participant would need to cope with the physical and psychological stress encountered in their daily lives. For this purpose, the minimum functional capacity required for each muscle group to minimize injury risk was calculated as the ideal target. In the exercise program, specialized isokinetic progressive resistance rehabilitation equipment called the EN-Dynamic system, developed by Enraf Nonius, was utilized. These devices incorporate both pneumatic and hydraulic systems and are designed to activate 13 distinct muscle groups. They provide objective measurements of muscle performance and allow resistance adjustments in increments as small as 0.5 kg. In this study, the maximum measurable output of each device served as a fixed reference point for the ideal maximum muscle capacity. These values were accepted as reference standards until more realistic benchmarks were developed. To allow for comparison of muscle group capacities among participants, each measured muscle value was calculated as a percentage of the device’s maximum measurement capability. This percentage was defined as the muscle functional capacity and was used in subsequent statistical analyses. Throughout this article, “muscle functional capacity” refers to the percentage of idealized performance capacity measured per muscle group using EN-Dynamic devices. This differs from general strength tests by offering muscle-specific, load-adjusted functional data. Unlike conventional gym equipment, the EN-Dynamic system was designed for use in the rehabilitation of older individuals and those with joint discomfort. Its smooth transitions and resistance behavior were suitable for individuals who could not tolerate shock loading and sudden joint movements. These features also made it possible to isolate each muscle group and apply precise force in a controlled manner. Additionally, the exercises were performed while seated, which reduced balance-related risks and allowed safe participation even for those with orthopedic conditions or balance disorders. To better illustrate the scope of targeted muscle training in this study, the 13 exercise devices and their corresponding muscle groups are listed in Table 1.

These devices were integrated with EN-Track software, which records muscle capacity based on the one-repetition maximum (1RM) test, tracks performance, and manages the exercise plan. However, EN-Track lacked the functionality required to generate exercise programs based on mathematical logic specific to each muscle group. To address this limitation and ensure more precise, personalized programming, a new software system was developed specifically for this study. This custom platform utilized a cross-linked algorithm to associate each participant’s medical history, daily activity level, psychological and physical stress scores, and the maximum functional capacity values for all 13 muscle groups. The result was an individualized and risk-sensitive training program optimized for safety and effectiveness, even in the presence of comorbidities.

### 2.7. Implementation and Monitoring

#### 2.7.1. Device Features

In this study, 13 specially developed devices using pneumatic resistance were employed to evaluate 13 different muscle groups. The pneumatic system allows the resistance level to be adjusted in 0.5 kg increments and maintains the same resistance throughout the range of motion. These devices provide a more controlled alternative to traditional weights for measuring muscle functional capacity, reduce the risk of injury during testing and exercise, and allow for precise adjustments tailored to the participant’s capabilities.

#### 2.7.2. Measurement Technique for Muscle Functional Capacity

The measurement process begins with the participant performing a movement using minimal pneumatic resistance. Resistance is then gradually increased until the participant is no longer able to initiate the movement. This value marks the end point of the test and is automatically recorded onto a smart chip card supplied by the manufacturer. The system is designed such that the evaluator cannot alter this data. The chip card is then scanned by the EN-Track software, which automatically transfers the test results to the database. To preserve data integrity, this software prohibits any manual modification of recorded values after upload.

#### 2.7.3. Data Handling and Software Integration

Once the measurements are recorded, the values are transferred to a custom-developed software platform designed by this study’s author. This program calculates the ratio of each measured value to the ideal reference capacity predefined for each device. The resulting percentages represent each participant’s maximum functional muscle capacity per muscle group and are displayed in a unified output format. The software also prevents any subsequent deletion or modification of these data to ensure full security and traceability.

#### 2.7.4. Adaptation for Athletes

For amateur or professional athletes, the resistance endpoint is defined differently. Rather than terminating the test when the participant can no longer initiate the movement, the measurement ends when the movement can no longer be completed. This approach reflects the performance expectations in athletic populations, where maximum output is prioritized. It allows the assessment protocol to adapt to both clinical and athletic demands.

#### 2.7.5. Clinical Utility and Report Generation

This measurement approach provides a detailed, group-specific evaluation of each muscle’s capacity to withstand daily physical and psychological stress. By incorporating factors such as comorbidities and individual stress levels, the system offers preliminary insight into which muscle groups or joints may be at elevated risk during daily activities. These insights form the basis of a personalized report on muscle functional capacity for each participant. To illustrate the practical application of this method, Table 2 presents a comparative test result from one participant (G.E., female, age 66), demonstrating the change in muscle functional capacity across a three-session period.

#### 2.7.6. Creation of the Exercise Program

The mathematical formula used in the study was developed into computer software. This software encompasses a mathematical algorithm based on cross-matching between 93 items and 23 options from the 4 questionnaires and the values obtained from 13 different muscle groups. Since the entire formula functions as software, it cannot be simply described in detail. The resistance level, number of sets, and number of repetitions for each of the 13 muscle groups are determined by this software through these cross-matching calculations. The resulting personalized exercise program is then transferred to the software system of the exercise devices (Table 3 and Table 4).

#### 2.7.7. Implementation of the Exercise Program

Each participant was provided with a personalized chip card containing their individual exercise plan, including the specific resistance levels, number of sets, and repetitions for each of the 13 targeted muscle groups. Participants initiated their sessions by inserting the chip card into the EN-Dynamic machines, which automatically adjusted the equipment settings according to the pre-programmed plan. Following each session, the chip card was scanned to detect any deviations or missed exercises. This allowed the system to flag inconsistencies and ensure strict adherence to the personalized protocol. In cases of inconsistency, participants were directed to repeat or correct incomplete sessions. This system functioned as a self-monitoring tool, enhancing the accuracy and consistency of exercise execution. The program was implemented three times per week, for a total of 36 sessions over a 12-week period. This frequency was chosen based on established findings in the literature, which suggest that three sessions per week offer an optimal balance between effectiveness and participant adherence. After completing all 36 sessions, participants underwent post-intervention measurements of muscle functional capacity and completed the same set of evaluation questionnaires administered prior to the program. These post-assessments enabled the evaluation of individual progress and the overall effectiveness of the personalized exercise method. Unlike subjective evaluations (e.g., “I feel better”) or conventional muscle tests that may vary based on the assessor or test conditions, this program utilized objective measurements. The EN-Dynamic system ensured consistency in test conditions, eliminating practitioner-related variability. As such, any post-program changes in measured values were considered real, reliable, and reflective of genuine functional improvements—independent of evaluator bias or participant perception.

The personalized exercise program for each muscle group, specifying the weights, repetitions, and sets, was meticulously generated through a sophisticated process:

- Negative factors affecting daily life, derived from the four administered questionnaires, were first converted into numerical coefficients.
- The ideal functional capacity each muscle needed to cope with daily life demands was then determined.
- Each muscle group’s current functional capacity was also converted into a corresponding coefficient.
- Disease-specific coefficients were formulated based on the disease severity questionnaire.
- Finally, these diverse coefficients interacted within a mathematical algorithm to precisely tailor the exercise prescription for each individual.

#### 2.7.8. Evaluation of Exercise Program Outcomes

To analyze the effectiveness of the intervention, both pre- and post-program data—including muscle functional capacity and questionnaire scores—were subjected to statistical analysis using SPSS 22.0 (Statistical Package for the Social Sciences) for Windows. The statistical methods included *t*-tests, one-way analysis of variance (ANOVA), Pearson correlation analysis, and Bonferroni multiple comparison tests.

## 3. Results

### 3.1. Participant Profile

Of the participants, 72.7% were female and 27.3% were male. The participants’ ages ranged from 16 to 94 years, with a mean age of 57.5 years. The age distribution was as follows: 29 individuals (17.16%) were in the <40 age group, 71 individuals (42.01%) were in the 40–64 age group, and 69 individuals (40.83%) were in the 65+ age group. The majority of participants (85%) had degenerative joint conditions and/or systemic diseases. Multiple comorbidities were present in many individuals. Additionally, 15% of the participants were otherwise healthy but reported experiencing fatigue during daily life activities (Table 5).

### 3.2. Muscle Functional Capacity Outcomes

**Objective:**

To examine the percentage change in muscle functional capacity before (pre-test) and after (post-test) the exercise program (Table 6 and Table 7).

Muscle functional capacity significantly increased following the exercise program, rising from a mean of 57.06% (±14.13) at pre-test to 75.17% (±12.19) at post-test. The paired-sample *t*-test revealed a highly significant result, t(168)=−30.65, p<0.01, with a very large effect size (Cohen’s d=−2.35).

Subgroup analyses also demonstrated consistent improvements across all gender and age groups. The mean gains ranged between 16.78% and 18.50%, with all results reaching statistical significance (p<0.0001) and Cohen’s *d* values between 1.84 and 2.60, indicating a robust and clinically meaningful impact. Importantly, even participants aged 65 and older achieved significant improvements, highlighting the feasibility of the program in older populations.

### 3.3. Combined Questionnaire Score

To evaluate broader psychosocial and functional changes, a combined score was derived from four questionnaires (Psychological Stress, Physical Stress, Quality of Life, and Disease Severity). Post-intervention analysis showed a significant decrease in the total combined score, from 142.08 (±23.37) at pre-test to 109.57 (±18.01) at post-test. This change was statistically significant, t(168) = 18.51, *p* < 0.01, with a very large effect size (Cohen’s d = 1.42) (Table 8).

Further breakdown by age and gender groups confirmed the consistency of these improvements. All subgroups experienced statistically significant score reductions with large to very large effect sizes (Cohen’s d ranging from 1.29 to 2.67). These findings support the generalizability of the intervention across demographic lines and reinforce its effectiveness in reducing psychological stress, physical strain, and perceived disease severity (Table 9).

### 3.4. Individual Questionnaire Outcomes

#### 3.4.1. Psychological Stress

A statistically significant reduction was observed in psychological stress levels following the exercise intervention (*p* < 0.01). The associated effect size (Cohen’s d = 1.25) indicates a large practical impact, suggesting that the personalized exercise method was highly effective in alleviating perceived psychological distress among participants (Table 10).

#### 3.4.2. Physical Stress

The analysis revealed a statistically significant decrease in physical stress scores from pre- to post-program assessments (*p* < 0.01). The effect size (Cohen’s d = 0.56) falls within the moderate range, indicating that the intervention contributed meaningfully to reducing physical fatigue and strain in daily life (Table 11).

#### 3.4.3. Quality of Life in Daily Activities

The statistically significant reduction in scores (*p* < 0.01) indicates an improvement in daily life quality, as lower scores reflect fewer limitations or complaints. The effect size (Cohen’s d = 0.75) suggests a moderate to large impact, supporting the practical relevance of the intervention for enhancing everyday functionality (Table 12).

#### 3.4.4. Disease Severity

Scores on the disease severity questionnaire significantly decreased after the intervention (*p* < 0.01), indicating symptom relief. The large effect size (Cohen’s d = 0.89) underscores the intervention’s clinical potential to reduce the burden of musculoskeletal and systemic conditions, even in individuals with chronic diagnoses (Table 13).

### 3.5. Gender-Based Comparisons

There was no statistically significant difference between male and female participants in the overall change in combined questionnaire scores (*p* = 0.97). The effect size was negligible, suggesting that the exercise program was equally effective for both genders (Table 14).

There was a statistically significant difference in the increase in muscle functional capacity between female and male participants (*p* = 0.01), with females showing greater gains (19.02% vs. 15.75%). However, the effect size (Cohen’s d: ≈0.44) indicates a small-to-moderate practical difference, suggesting that while the effect is statistically meaningful, the actual magnitude of the difference may not be clinically substantial. This discrepancy may be partially explained by the lower baseline capacity observed in female participants (Table 15).

None of the four questionnaire domains showed statistically significant differences in pre–post improvement between male and female participants (*p* > 0.05 for all comparisons). These findings suggest that the personalized exercise program produced comparable psychosocial and clinical benefits across genders (Table 16).

### 3.6. Age-Based Comparisons

In this study, participants were divided into three age groups:

- Under 40 years;
- 40–64 years;
- 65 years and older.

While older participants appeared to show slightly greater improvements in muscle functional capacity, the differences across age groups did not reach statistical significance (*p* = 0.10). These findings suggest that the exercise intervention was similarly effective across age categories, supporting its applicability across the adult lifespan (Table 17).

No statistically significant differences were found in total questionnaire score improvements across age groups (*p* = 0.56). This suggests that the exercise program led to comparable psychosocial and functional gains across all age brackets, reinforcing its broad applicability regardless of age (Table 18).

Participants under the age of 40 exhibited significantly smaller improvements in physical stress scores compared with the 40–64 group (*p* = 0.03). This outcome is likely attributable to their relatively higher baseline muscle functional capacity, which may have served as a protective factor against the physical demands of daily life. This finding aligns with previous results indicating greater initial capacity and lower baseline stress levels in younger individuals. In contrast, participants aged 40 and above—starting from higher levels of physical burden—showed more pronounced reductions in stress following the intervention. These results highlight the particular effectiveness of the exercise program in midlife and older populations who may be more susceptible to physical fatigue (Table 19).

Differences in improvement levels on the Physical Stress questionnaire were significantly influenced by age. Participants under 40 experienced smaller reductions in stress, likely due to their better baseline muscle function and lower initial fatigue. Meanwhile, the 40–64 and 65+ groups exhibited more marked improvements, supporting the view that individuals with higher baseline stress benefit more visibly from targeted intervention.

For the remaining questionnaires (psychological stress, quality of life, and disease severity), no statistically significant differences in improvements were found across age groups (*p* > 0.05), suggesting that the benefits of the intervention in these domains were comparable across all age brackets.

### 3.7. Correlation Analyses

This section reports Pearson correlation analyses between the increase in muscle functional capacity (i.e., percentage difference between pre- and post-tests) and the reductions in questionnaire scores (pre-test minus post-test scores) (Table 20).

Since all *p*-values are greater than 0.05, none of the correlations were statistically significant. In other words, no linear relationship was detected between improvements in muscle functional capacity and reductions in questionnaire scores. This suggests that the relationship between physical improvements and perceived psychological or disease-related improvements may not follow a uniform correlation pattern. Variability in participants’ medical profiles, sources of stress, and daily life burdens may contribute to the weak or neutral correlations observed.

#### 3.7.1. Gender-Based Correlation Patterns

Similarly to the overall sample, no statistically significant correlations were found between muscle functional capacity improvements and questionnaire score changes when analyzed separately for males and females (all *p* > 0.05).

#### 3.7.2. Age-Specific Correlations

In the age group under 40 years, a moderate negative correlation was observed between improvements in muscle functional capacity and reductions in disease severity scores (r = −0.418, *p* = 0.02). This indicates that younger participants with better baseline muscle capacity were more likely to experience meaningful reductions in disease burden.

These individuals had initial muscle functional capacity levels that were already sufficient to support daily activities, and their disease scores were also relatively low. This balance likely contributed to a more detectable correlation between small increases in muscle capacity and noticeable reductions in disease burden.

In contrast, participants in the 40–64 and 65+ age groups showed no statistically significant correlations between muscle functional capacity gains and questionnaire score changes. This may be due to the decline in baseline functional capacity and an increase in disease burden with age, making the relationship between strength improvement and perceived well-being less directly proportional. These findings reinforce the importance of improving muscle functional capacity, especially in older adults, where even substantial strength gains may not immediately translate into perceptual improvements due to higher initial limitations.

#### 3.7.3. Interpretation of Correlation Findings

The absence of strong linear correlations in the overall sample highlights the multifactorial nature of perceived well-being and its complex relationship with physical improvements. This suggests that the relationship between improvements in muscle functional capacity and perceived psychological or disease-related improvements may not be linear or consistent. Nevertheless, moderate correlations were identified in specific subgroups—particularly among participants under the age of 40.

To better understand this variability, subgroup-level findings offer additional insight. At baseline, younger participants exhibited lower levels of physical stress, which may be attributed to their higher initial muscle functional capacity. According to the results of the Quality of Daily Life Activities questionnaire, a statistically significant improvement (i.e., a reduction in score) was observed only in the <40 age group (p<0.05). In contrast, participants in the 40–64 and 65+ age groups did not show significant post-intervention changes in this domain (p>0.05).

While muscle functional capacity improved in both of the older age groups—from a mean of 58.65% ± 11.26 to 78.78% ± 7.89 in the 40–64 group and from 51.21% ± 12.18 to 68.00% ± 12.09 in the 65+ group—these values remained below the 80% functional capacity threshold considered ideal for independent, high-quality daily functioning (see Table 7). This may explain the absence of statistically significant improvements in perceived quality of life, despite measurable physical gains.

These findings indicate that higher functional capacity—likely above 80%—may be required to produce noticeable improvements in daily quality of life. For older adults, this threshold might only be reached with longer-term training interventions.

Moreover, it is possible that the existing version of the Quality of Life questionnaire lacked sufficient sensitivity to detect subtle yet meaningful changes—particularly in older adults. Future large-scale implementations of this program may benefit from revised or newly developed tools that better capture multidimensional improvements in physical, emotional, and functional domains.

### 3.8. Summary of Key Findings

This section summarizes the key findings from the study and provides an integrated interpretation of the intervention’s impact across physiological, psychological, and functional domains.

#### 3.8.1. Functional Capacity Gains

A substantial and statistically highly significant increase in muscle functional capacity was observed, rising from 57.06% at the pre-test to 75.17% at the post-test. This improvement was associated with a very large effect size (Cohen’s d > 2).

#### 3.8.2. Questionnaire Improvements

Statistically significant improvements (score reductions) were found in all four questionnaires—Psychological Stress, Physical Stress, Quality of Daily Life Activities, and Disease Severity—when comparing pre-test and post-test scores. The effect sizes of these improvements ranged from moderate to large (Cohen’s d = 0.56 to 1.25).

#### 3.8.3. Gender and Age Group Findings

While male and female participants showed similar levels of improvement in questionnaire scores, the average increase in muscle functional capacity was slightly higher in females (19.02 points) than in males (15.75 points).

No statistically significant differences were found in muscle functional capacity gains or questionnaire improvements across the three age groups: <40 years, 40–64 years, and 65+ years. However, due to the relatively lower baseline physical stress levels in the under-40 group, the amount of stress reduction was correspondingly lower.

#### 3.8.4. Correlation Analyses

**Figure 1:**
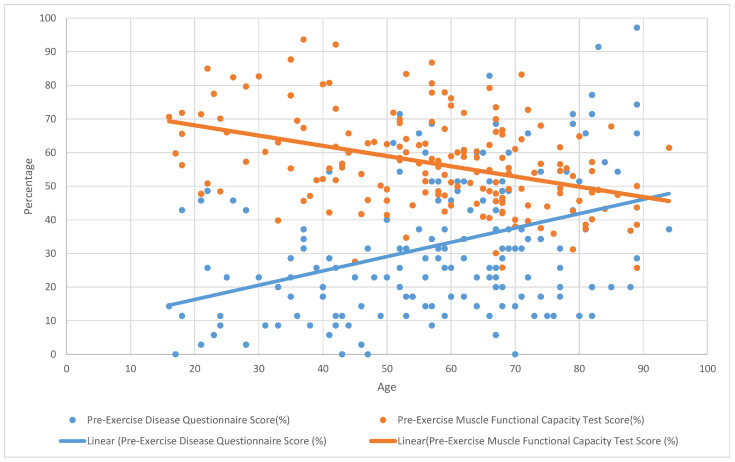
The correlation between pre-exercise muscle functional capacity and disease questionnaire scores.

**Figure 2:**
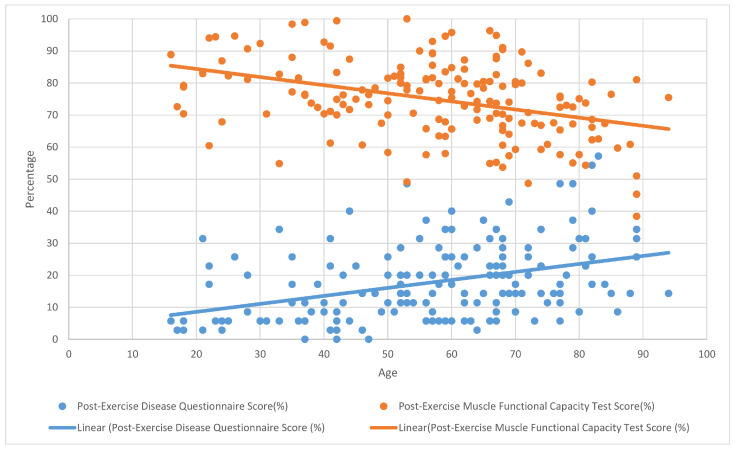
The correlation between post-exercise muscle functional capacity and disease questionnaire scores.

No statistically significant correlation was found between muscle functional capacity improvement and questionnaire score reductions in the overall participant group. However, a moderate negative correlation was identified in the under-40 age group between disease severity score and muscle functional capacity improvement (r = −0.418, *p* = 0.02) (see Figure 1 and Figure 2).

#### 3.8.5. Integration of Results

In summary, the personalized exercise intervention significantly enhanced muscle functional capacity and was associated with notable improvements in psychological stress, physical stress, disease burden, and perceived quality of daily life. These effects were consistently observed across both genders and all age groups. While females showed slightly higher strength gains, the overall outcomes suggest that the program is effective and adaptable regardless of age or health condition. The absence of strong correlations between physical and subjective outcomes in the full sample reflects individual variability and highlights the complexity of translating physical improvements into perceptual changes.

#### 3.8.6. Additional Notes

- Statistical Significance (*p*-values):
  All *p*-values presented in the tables and the *t*-test results reflect the reliability of the statistical analyses. For example, a *p*-value of <0.01 indicates that the result is statistically significant at the 99% confidence level.
- Cohen’s d Interpretation (Effect Size):
  - Around 0.2 = small effect;
  - Around 0.5 = moderate effect;
  - 0.8 and above = large effect;
  - 1.2 and above = very large effect.
- Negative Correlation (r<0):
  Although an inverse relationship between increasing muscle functional capacity and decreasing questionnaire scores is expected, it may not be consistently observed across all individuals due to personal differences in stress profiles, daily life demands, or health status. Therefore, correlations may appear weak or neutral in the general population.
- Statistical Methods: All analyses were based on the study dataset and conducted using the following:
  - Paired-sample *t*-tests for pre–post comparisons;
  - Independent-sample *t*-tests or one-way ANOVA for gender and age group comparisons;
  - Pearson correlation coefficients for correlation analyses.

## 4. Discussion

The global population is aging at a rapid pace [3], leading to a significant increase in the healthcare needs and costs associated with older adults [18]. Numerous studies indicate that maintaining physical activity is essential for preserving quality of life in older individuals, with structured exercise being identified as a central component of healthy aging [8,15,19].

In addition to the natural aging process, physical and psychological stress—as well as overuse of muscles—have been shown to contribute to declines in muscular strength [8,20,21]. Both inactivity and repetitive strain can result in muscle weakening, eventually leading to sarcopenia. As muscle strength diminishes, individuals often become more sedentary, further accelerating physical decline and withdrawal from daily activities [22]. Evidence also supports that regular exercise mitigates the risk of developing sarcopenia as individuals age [23]. Moreover, even in younger individuals without prior joint issues, excessive sports or physical activity can lead to long-term joint degeneration and a decline in functional quality of life [24,25,26].

Beyond mechanical or activity-related stressors, biological aging mechanisms such as chronic psychological stress and pain may also contribute to muscular degeneration. For instance, chronic stress has been linked to telomere shortening, a biomarker of cellular aging, which is in turn associated with reduced muscle strength, as demonstrated by Sibille [27]. While exercise is commonly prescribed to alleviate chronic pain, improper loading before reaching an adequate level of functional capacity can increase the risk of exacerbating pain rather than relieving it.

Although exercise has consistently been shown to enhance muscle strength and improve quality of life in older populations, programs that apply generic or high-intensity loading strategies may inadvertently cause musculoskeletal harm. Therefore, a growing body of research now supports the need for individualized exercise plans that consider personal thresholds and health conditions [28].

Standardized exercise regimens often exceed the capabilities of older adults, many of whom live with chronic conditions or joint limitations [22,25]. This frequently results in reluctance to engage in physical activity due to fear of injury or worsening pain. To avoid excessive joint loading and ensure safe participation, personalized program design is essential [22,29]. As existing models often fall short in supporting a sustainable return to active living, there is an urgent need for innovative and adaptable approaches [19].

In response to this gap, the present study aimed to evaluate whether a personalized exercise program based on muscle functional capacity could significantly improve both objective muscular performance and subjective quality of life across different age groups. To support this aim, we first introduced and operationalized the concept of muscle functional capacity as a measurable, percentage-based value indicating the adequacy of strength in 13 targeted muscle groups. Although previous research has explored various strength training modalities, a universally applicable exercise protocol that enables pain-free, independent performance of daily tasks has not been firmly established. This highlights the ongoing need to refine the concept of functional capacity. In our study, we addressed this need by developing a measurement protocol capable of evaluating the capacity of individual muscle groups independently. These results were then compared with ideal thresholds and used to generate personalized exercise programs tailored to each participant.

Because each muscle group possesses its own unique functional capacity, individuals require structured and targeted guidance to achieve optimal activation levels. This is particularly critical for individuals with joint-related or systemic conditions who may be hesitant to engage in physical activity due to concerns about safety or symptom exacerbation. The personalized exercise model developed in this study was specifically designed to address these concerns by calibrating resistance and repetition parameters to individual muscular needs.

In addition to individualizing the intervention, our standardized approach allowed for statistically valid comparisons across participants and subgroups. Recognizing the considerable role of physical and psychological stress in musculoskeletal overload and degeneration, we incorporated mathematical modeling and validated survey instruments to quantify physical strain and mental fatigue (see Supplementary Materials S1–S4). These tools were central to assessing the broader impact of the intervention beyond muscle capacity alone.

Our findings demonstrated a statistically significant improvement in muscle functional capacity (t = –30.65, *p* < 0.01), along with meaningful reductions in psychological stress (t = 16.35, *p* < 0.01), physical stress, disease burden, and limitations in daily activities. Notably, however, no statistically significant correlation was found between improvements in muscle functional capacity and changes in quality of life scores (r = 0.06, *p* = 0.47), despite participants reporting subjective improvements in stress levels.

This disconnect between objective physical gains and subjective quality of life measures suggests that perceived well-being may not align linearly with measurable physiological changes. Such divergence likely reflects individual variability in psychological baseline, stress perception, health literacy, and expectations. It also underscores the limitations of conventional measurement tools in capturing nuanced psychosocial effects of exercise interventions. Future research should consider integrating refined or alternate assessment tools with increased sensitivity—particularly for use in older populations.

Furthermore, we believe that the current version of the Quality of Daily Life Activities questionnaire may not adequately reflect age-specific functional contexts and priorities. Its structure may lack the granularity required to detect subtle but clinically important changes in different demographic subgroups. Additionally, a three-month intervention period, while sufficient to observe muscular gains, may not be long enough to elicit comprehensive improvements in daily functioning. Future studies employing longer training schedules may better elucidate the timeline required to achieve lasting changes in subjective quality of life.

Beyond individual outcomes, these findings carry broader implications for healthcare systems. It is well established that medical costs rise in parallel with the severity of chronic illness and physical dependency [30]. Therefore, effective interventions must consider not only the presence of musculoskeletal or systemic disorders but also their intensity and impact on daily living. Our study emphasizes the importance of evaluating disease severity alongside stress exposure and activity levels, using quantifiable tools to track shifts in clinical burden and guide resource allocation.

A notable limitation of previous research is its predominant focus on healthy older adults or younger populations, often excluding individuals with comorbidities or reduced functional capacity [10]. In contrast, the present study included participants from diverse age groups and with varying health conditions, aiming to evaluate how a personalized exercise program could affect both disease severity and muscle functional capacity. Our findings suggest that improvements in muscle strength may be associated with reductions in disease burden, particularly among older adults.

Prior work by Briggs et al. reported that women are more likely than men to be hospitalized due to joint-related conditions, primarily due to the lower baseline muscle strength in females [25]. In our sample, women had a pre-intervention average muscle functional capacity of 51.92%, a level sufficient for performing basic daily tasks without considerable strain. Following the intervention, this value rose to 70.95%, indicating a moderate tolerance to physical stress. This improvement aligns with the positive shifts observed in their questionnaire scores, suggesting both functional and perceptual gains.

Similarly, male participants demonstrated a baseline muscle functional capacity of 70.22%, corresponding to moderate-intensity task performance. After the exercise program, this increased to 85.97%, indicating the capacity to manage high-intensity physical demands. These improvements suggest that individualized, capacity-based training can reliably produce functional gains across genders. Importantly, they also support the concept that a minimum threshold of muscle functional capacity may be required to sustain physical independence.

Notably, participants aged 65 and older—while exhibiting meaningful improvements—did not reach the 80% functional capacity threshold that is often considered necessary for high-quality, independent daily functioning. This may explain why perceived improvements in quality of life were less pronounced in this group compared to their younger counterparts. Our findings reinforce the hypothesis that this 80% threshold may serve as a critical inflection point—one at which physical improvements begin to translate into more noticeable psychological and functional benefits.

Collectively, these outcomes align with and expand upon the findings of Briggs et al., emphasizing the clinical importance of strengthening interventions tailored to individual needs—especially among older adults—to mitigate disease severity and promote physical resilience [25].

Specifically, our data showed significant muscle functional capacity gains in both male and female participants. Among women and men, the average pre-to-post strength increases were 19.02% ± 7.78 and 15.75% ± 7.12, respectively, (t=−2.53, p=0.01; see Table 6). The questionnaire scores also improved substantially, with a decrease from 142.08 ± 23.37 at baseline to 109.57 ± 18.01 post-intervention (t=18.51, p<0.01; Cohen’s d=1.42; see Table 8). These findings confirm that the personalized exercise model produced consistent and clinically meaningful outcomes across both gender and age categories (see Table 7 and Table 9).

The exceptionally large effect sizes observed in this study (e.g., Cohen’s d>2.0) indicate a strong potential for the intervention to enhance muscle functional capacity, particularly in individuals with low baseline strength. These unusually strong effects are likely the result of multiple contributing factors, including the initially diminished functional capacity—especially among older participants—and the high measurement precision provided by the EN-Dynamic system. This system minimizes inter-rater variability and standardizes resistance application, offering a consistent and objective assessment across sessions and participants. While these results are highly promising, effect sizes of this magnitude should be interpreted cautiously. Replication in larger, more diverse clinical populations is necessary to validate generalizability.

Beyond physical outcomes, enhancing participant motivation—particularly among older adults—has been identified as a critical factor in sustaining engagement in exercise interventions [31]. Providing individuals with clear, personalized feedback regarding their progress can support adherence and psychological investment. In this study, each participant received an individualized functional capacity report for all 13 targeted muscle groups. These reports allowed participants to objectively monitor their advancement toward predefined functional thresholds associated with safe and independent living.

A key objective of the study was to assess whether improvements could be achieved consistently across different age groups, including adults aged 65 and older. This demographic is often underrepresented in exercise research due to concerns about feasibility, safety, and compliance. Remarkably, all 169 participants, including those over 65, completed the 12-week program without medical or procedural complications. This high adherence rate demonstrates the practicality, safety, and accessibility of the personalized exercise method, reinforcing its suitability for diverse clinical and community settings.

### 4.1. Study Limitations

This study investigated the effects of a personalized exercise program on muscle functional capacity and quality of life in daily activities. However, several methodological limitations should be acknowledged to contextualize the findings.

#### 4.1.1. Lack of a Control Group

A non-exercising control group was not included in this initial phase of the study. This decision was based on the established consensus in the literature that measurable improvements in muscle functional capacity do not occur in the absence of structured physical activity. Furthermore, no current evidence suggests that strength gains can emerge without targeted exercise. Thus, the focus of this phase was on defining the methodology and assessing its practical application in diverse populations, rather than on comparative efficacy. Nonetheless, the long-term retention of exercise-related improvements remains a relevant research question. To address this, participants were instructed to avoid other forms of structured exercise for one year following the program. A follow-up study has been initiated to assess the persistence of functional gains by comparing previously active participants with a newly recruited non-exercise control group who underwent baseline testing and will be re-assessed after one year. This follow-up is designed to explore long-term trends in sarcopenia, examine strength trajectories under different health profiles, and further validate the sustainability of the intervention.

#### 4.1.2. Absence of Direct Comparison with Traditional Exercise Methods

While this study included participants who had previously discontinued traditional exercise programs due to pain, comorbidities, or perceived inefficacy, no formal comparative analysis was performed between the personalized method and standard exercise protocols. A comparative evaluation will be addressed in a future phase of the research, which aims to examine the differential outcomes, safety profiles, and participant adherence rates between conventional and personalized approaches. Overall, the current study should be regarded as a pilot investigation focused on method validation and feasibility. More comprehensive comparative analyses and long-term evaluations are planned in subsequent stages of this research program.

## 5. Conclusions

The findings of this study provide compelling evidence that a personalized exercise protocol—calibrated using objective measurements across 13 distinct muscle groups—can significantly enhance muscle functional capacity in a clinically diverse population. The intervention also yielded substantial reductions in psychological and physical stress, disease severity, and limitations in daily life activities, with consistent benefits observed across different age groups and both genders. Personalized resistance-based exercise may be especially beneficial for older adults, supporting their return to an active lifestyle and potentially reversing early signs of sarcopenia. The structured and individualized nature of the protocol contributed to high adherence and demonstrated feasibility even among participants aged 65 and older, all of whom completed the program without complications. Beyond its clinical implications, the study offers a methodological contribution through the direct, group-specific measurement of muscle functional capacity. This approach enables more precise tracking of physical rehabilitation and opens new avenues for future research examining biological markers such as telomere length and mitochondrial adaptation in response to exercise.

## Figures and Tables

**Table 1:** The 13 muscle groups and the corresponding exercise devices used in the program.

Device Name	Targeted Muscle Group
Leg Press	Hip and knee muscles
Chest Press	Chest and anterior shoulder muscles
Leg Extension	Anterior thigh muscles
Rowing	Back and neck muscles
Triceps	Posterior arm muscles
Back Trainer	Lumbar and spinal muscles
Abdominals	Abdominal muscles
Lat Pulldown	Shoulder and arm muscles
Leg Curl	Posterior thigh muscles
Fly	Anterior chest muscles
Adduction	Hip and leg adductor muscles
Abduction	Hip and leg abductor muscles
Shoulder Press	Shoulder and arm muscles

In the first column of the table, the names of the devices are shown, and in the second column, the corresponding muscle groups are indicated. This list only defines the muscle groups included in the study.

**Table 2:** Comparative muscle functional capacity test during exercise.

Muscle Groups	2 December 2021 Max. Functional Capacity (%)	26 April 2022 Max. Functional Capacity (%)	Development (% Increase)
Leg Press	85.00	100.00	15.00
Chest Press	35.00	58.33	23.33
Leg Extension	66.00	75.00	9.00
Rowing	53.00	90.00	37.00
Triceps	52.00	73.00	21.00
BackTrainer	71.25	100.00	28.75
Abdomen	53.75	100.00	46.25
Pull Down	57.50	75.00	17.50
Leg Curl	78.57	100.00	21.43
Fly	27.69	32.31	4.62
Adduction	66.67	80.00	13.33
Abduction	91.67	100.00	8.33
Shoulder	56.67	63.33	6.66
**Total Average**	61.14	80.54	19.40

Test Date: 26 April 2022; Name: G. E.; Age: 66; Gender: Female.

**Table 3:** An example of an exercise program according to the first muscle functional capacity test.

Muscle Group	Set 1	Reps 1	Resistance 1 (kg)	Set 2	Reps 2	Resistance 2 (kg)
Leg Press	2	16	25.50	2	16	30.60
Chest Press	2	22	6.30	2	22	7.56
Leg Extension	2	28	9.90	2	28	11.88
Rowing	2	27	7.95	2	27	9.54
Triceps	2	27	7.80	2	27	9.36
Back Trainer	3	23	8.55	3	23	10.26
Abdomen	3	22	6.45	3	22	7.74
Pull Down	3	22	6.90	3	22	8.28
Leg Curl	3	26	8.25	3	26	9.90
Fly	3	26	2.70	3	26	3.24
Adduction	3	29	6.00	3	29	7.20
Abduction	3	30	8.25	3	30	9.90
Shoulder	3	29	5.10	3	29	6.12

Test Date: 26 April 2022; Name: G. E.; Age: 66; Gender: Female.

**Table 4:** An example of an exercise program according to the last muscle functional capacity test.

Muscle Group	Set 1	Reps 1	Resistance 1 (kg)	Set 2	Reps 2	Resistance 2 (kg)
Leg Press	2	17	30.00	2	17	36.00
Chest Press	2	23	10.50	2	23	12.60
Leg Extension	2	28	11.25	2	28	13.50
Rowing	2	29	13.50	2	29	16.20
Triceps	2	28	10.95	2	28	13.14
Back Trainer	3	24	12.00	3	24	14.40
Abdomen	3	24	12.00	3	24	14.40
Pull Down	3	23	9.00	3	23	10.80
Leg Curl	3	26	10.50	3	26	12.60
Fly	3	26	3.15	3	26	3.78
Adduction	3	30	7.20	3	30	8.64
Abduction	3	30	9.00	3	30	10.80
Shoulder	3	29	5.70	3	29	6.84

Test Date: 26 April 2022; Name: G. E.; Age: 66; Gender: Female.

**Table 5:** Diseases of the participants.

Disease	Participants (n, %)
Lumbar Disc Disease	104 (61.53)
Surgery for Lumbar Disc	11 (6.50)
Cervical Disc Disease	37 (21.99)
Surgery for Cervical Disc Disease	2 (1.18)
Shoulder Problems	34 (20.11)
Hip Problems	26 (15.38)
Knee Problems	49 (28.99)
Hypertension	23 (13.60)
Diabetes Mellitus	21 (12.42)
Obesity	14 (8.28)
Mild Neuropathy	16 (9.46)
Hypothyroidism	11 (6.50)
COVID-19	2 (1.18)
Cancer (older than two years)	10 (5.95)

Some participants have more than one disease.

**Table 6:** Muscle functional capacity (pre–post comparison).

Test Period	Mean (%)	Standard Deviation (±SD)	t(df), *p*	Cohen’s d
Pre-Test	57.06	±14.13	t(168) = −30.65, *p* < 0.01	−2.35
Post-Test	75.17	±12.19		Very large effect size

Statistical Method: Paired-Sample *t*-test.

**Table 7:** Muscle functional capacity by gender and age group.

Group	Pre Mean ± SD	Post Mean ± SD	Δ (Mean ± SD)	t(df)	*p*	Cohen’s d
Women < 40	59.31 ± 10.16	76.62 ± 8.50	17.31 ± 6.85	11.30 (19)	<0.0001	2.35
Women 40–64	56.12 ± 13.17	73.84 ± 11.98	17.72 ± 8.69	16.18 (55)	<0.0001	2.04
Women 65+	53.89 ± 14.10	70.67 ± 12.12	16.78 ± 9.11	11.65 (46)	<0.0001	1.84
Men < 40	63.25 ± 9.88	81.75 ± 10.12	18.50 ± 7.12	7.80 (8)	<0.0001	2.60
Men 40–64	56.78 ± 13.45	74.22 ± 12.35	17.44 ± 7.65	11.40 (14)	<0.0001	2.28
Men 65+	55.50 ± 12.50	72.80 ± 11.00	17.30 ± 7.00	11.62 (21)	<0.0001	2.47

Effect size interpretation based on Cohen’s d: 0.2 = small, 0.5 = moderate, 0.8+ = large, 1.2+ = very large.

**Table 8:** Combined questionnaire score (pre–post comparison).

Test Period	Mean (Total Score)	SD (±)	t(df), *p*	Cohen’s d
Pre-Test	142.08	23.37	t(168)=18.51, p<0.01	1.42
Post-Test	109.57	18.01		Very large effect size

Statistical Method: Paired-Sample *t*-test.

**Table 9:** Combined questionnaire score by gender and age group.

Group	n	Pre Mean ± SD	Post Mean ± SD	Δ (Mean ± SD)	t(df), *p*	95% CI, d
Female < 40	20	149.08 ± 11.48	127.31 ± 10.68	21.77 ± 8.96	10.87 (19), p<0.0001	[17.58, 25.96], d = 2.43
Female 40–64	56	142.80 ± 24.00	107.10 ± 19.00	35.70 ± 24.50	11.55 (55), p<0.0001	[29.53, 41.87], d = 1.46
Female ≥65	47	144.50 ± 23.50	108.20 ± 18.50	36.30 ± 22.00	9.63 (46), p<0.0001	[29.14, 44.64], d = 1.65
Male < 40	9	151.33 ± 10.19	130.00 ± 10.10	21.33 ± 7.98	8.01 (8), p<0.0001	[16.99, 27.47], d = 2.67
Male 40–64	15	144.20 ± 23.82	108.76 ± 17.43	35.44 ± 25.09	7.06 (14), p<0.0001	[25.08, 45.80], d = 1.41
Male ≥65	22	138.50 ± 26.50	105.10 ± 19.00	33.40 ± 26.00	6.03 (21), p=0.0010	[20.40, 46.40], d = 1.29

**Table 10:** Psychological stress scores (pre–post comparison).

Test Period	Mean ± SD	t(df), *p*	Cohen’s d
Pre-Test	31.12 ± 9.31	t(168)=16.35, p<0.01	1.25
Post-Test	18.79 ± 6.40		Large effect size

Statistical Method: Paired-Sample *t*-test.

**Table 11:** Physical stress scores (pre–post comparison).

Test Period	Mean ± SD	t(df), *p*	Cohen’s d
Pre-Test	48.27 ± 8.62	t(168)=7.24, p<0.01	0.56
Post-Test	43.94 ± 6.41		Moderate effect size

Statistical Method: Paired-Sample *t*-test.

**Table 12:** Quality of life scores (pre–post comparison).

Test Period	Mean ± SD	t(df), *p*	Cohen’s d
Pre-Test	51.54 ± 12.30	t(168)=9.77, p<0.01	0.75
Post-Test	40.75 ± 9.49		Moderate to large effect size

Statistical Method: Paired-Sample *t*-test.

**Table 13:** Disease severity scores (pre–post comparison).

Test Period	Mean ± SD	t(df), *p*	Cohen’s d
Pre-Test	11.30 ± 7.14	t(168)=11.61, p<0.01	0.89
Post-Test	6.27 ± 4.07		Large effect size

Statistical Method: Paired-Sample *t*-test.

**Table 14:** Combined questionnaire score difference by gender.

Group	Mean Difference ± SD	t(df), *p*	Cohen’s d
Male	32.42 ± 19.70	t(169)=−0.03, p=0.97	≈0
Female	32.54 ± 24.19		Negligible effect

Statistical Method: Independent-Sample *t*-test.

**Table 15:** Increase in muscle functional capacity by gender.

Group	Mean Difference ± SD	t(df), *p*	Cohen’s d
Male	15.75 ± 7.12	t(169)=−2.53, p=0.01	≈0.44
Female	19.02 ± 7.78		Small to moderate effect

Statistical Method: Independent-Sample *t*-test.

**Table 16:** Questionnaire subscore changes by gender.

Questionnaire	Female Mean Δ	Male Mean Δ	t-Value	*p*-Value	Significance
Psychological Stress	12.89	10.80	−1.24	0.22	Not significant
Physical Stress	4.63	3.54	−0.80	0.42	Not significant
Quality of Life in Activities	10.15	12.50	0.95	0.35	Not significant
Disease Severity	4.87	5.43	0.58	0.56	Not significant

Includes all four questionnaires: Psychological Stress, Physical Stress, Quality of Life, and Disease Severity.

**Table 17:** Muscle functional capacity gain by age group.

Age Group	Mean Difference (%)	Standard Deviation
<40 years	∼15.52	∼7.09
40–64 years	∼18.32	∼7.32
65+ years	∼19.17	∼8.23

Statistical Method: One-Way ANOVA. ANOVA Result: F=2.32, p=0.10.

**Table 18:** Combined questionnaire score difference by age group.

Age Group	Mean Difference ± SD (Total Score)
<40 years	36.60 ± 25.73
40–64 years	31.87 ± 25.22
65+ years	31.40 ± 19.14

Statistical Method: One-Way ANOVA. ANOVA Result: F=0.58, p=0.56.

**Table 19:** Physical stress score difference by age group.

Age Group	Physical Stress Mean Difference
<40 years	0.93
40–64 years	5.35
65+ years	4.71

Statistical Method: One-Way ANOVA with Post hoc Analysis. ANOVA Result: F=3.57, p=0.03; Post hoc Result: Significant difference observed between the <40 and 40–64 age groups.

**Table 20:** Correlations between muscle functional capacity gains and questionnaire improvements.

Variables	Correlation (r)	*p*-Value
Muscle Functional Capacity Increase ∼ Total Questionnaire Score Difference	0.02	0.79
Muscle Functional Capacity Increase ∼ Psychological Stress Difference	−0.02	0.82
Muscle Functional Capacity Increase ∼ Physical Stress Difference	0.02	0.83
Muscle Functional Capacity Increase ∼ Quality of Life Difference	0.06	0.47
Muscle Functional Capacity Increase ∼ Disease Severity Difference	−0.05	0.54

Statistical Method: Pearson Correlation Analysis.

## Data Availability

The datasets generated and/or analyzed during the current study are not publicly available due to concerns about compromising individuals’ private data/information, but they are available from the corresponding author upon reasonable request.

## Supplementary Materials

The following supporting information can be downloaded at: https://www.mdpi.com/article/10.3390/ijerph22091344/s1, Supplementary Material S1: Modified Psychological Stress Test. Supplementary Material S2: Daily Life Quality Level Test. Supplementary Material S3: Physical Stress Test. Supplementary Material S4: Disease Questionnaire.
