# Supplementary material for: The Effect of a Personalized Exercise Program on Muscle Functional Capacity and Quality of Daily Life: A Randomized Pilot Study
Source: Int J Environ Res Public Health. 2025 Aug 28;22(9):1344. doi: 10.3390/ijerph22091344 (PMC12469410; doi:10.3390/ijerph22091344)
Supplement: Supplementary file 1 [file ijerph-22-01344-s001.zip › ijerph-3687083-SM.pdf]

Supplementary Materials: The Effect of a Personalized Exercise Program on Muscle Functional Capacity and Quality of Daily Life: A Randomized Pilot Study

Semra Ercan *,†, Türkü Yalçınol † and Özge Öngel †

Supplementary Material S1: Modified Psychological Stress Test

This test was modified from the 9-item scale developed by Lemyre and Tessier. Participants were asked to choose the response that best reflected their experience over the past month for each statement below:

- I feel calm
- I feel rushed
- I have physical aches (Neck, head, stomach, etc.)
- I feel preoccupied, stuck, or worried
- I feel confused, I lack concentration, I cannot focus
- I feel full of energy and desire
- I feel a great weight on my shoulders
- I have difficulty controlling my reaction
- I feel stressed

Each item was scored using the following eight-point Likert scale: *Not at all, Not Really, Very Little, A Bit, Quite A Bit, A Lot, Very Much, Extremely.*

Supplementary Material S2: Daily Life Quality Level Test

Instruction: Provide the closest answer to your experience in the last month.

- **How often do you experience physical pain and discomfort?**
  Not at all, Rare, On stressful days, On tired days, Often
- **How easily do you get tired?**
  Not at all, Rare, On stressful days, Often, Even simple tasks
- **How satisfied are you with your quality of life?**
  Very much, A lot, Somewhat, A bit, Not at all
- **Sleep and rest:**
  Normal, Difficulty falling asleep, Frequent waking at night, Waking tired, Can only sleep towards morning
- **How would you rate your memory?**
  Very good, Good, Somewhat, Weak, Too weak
- **Lack of positive emotions:**
  Not at all, Little, A bit, A lot, Very much
- **How good is your concentration?**
  Very much, More, Somewhat, Little, Very little
- **Low self-esteem:**
  Not at all, Little, A bit, A lot, Very much
- **Body image and appearance dissatisfaction:**
  Not at all, Little, A bit, A lot, Very much
- **How worried are you?**
  Not at all, Little, A bit, A lot, Very much

- **How do difficulties in moving affect your life flow?**
  No strain, Hard on stressful days, Get tired quickly, Difficult doing what I used to, Can only do simple personal tasks
- **Intensity of daily life activity:**
  Not at all, Little, A bit, A lot, Very much
- **Addiction to medical substances/aids:**
  No use, Occasionally uses analgesics, Occasionally uses antihypertensives, Dependent on less than 4 drugs, Dependent on more than 4 drugs
- **Addiction to non-medical substances (alcohol, tobacco, drugs):**
  Not at all, Little, A bit, A lot, Very much
- **Can you do your work without difficulty?**
  Very good, Good, Somewhat, Bad, Very bad
- **How would you rate your working capacity?**
  Very good, Good, Somewhat, Bad, Very bad
- **How long can you be alone in your life?**
  Can stay alone, Afraid of being alone, Afraid of future loneliness, Needs family help, Must live with someone
- **How concerned are you about your safety and security?**
  Not worried, Worried about home accidents, Fear of falling in street, Need help on street, Cannot stay alone
- **How much does sadness/depression affect your daily life?**
  Not at all, Little, A bit, A lot, Very much
- **Freedom/need for physical security:**
  Not at all, Little, A bit, A lot, Very much
- **Home surroundings (lack of comfort):**
  Not at all, Little, A bit, A lot, Very much
- **Lack of job satisfaction:**
  Not at all, Little, A bit, A lot, Very much
- **Insufficient financial resources:**
  Not at all, Little, A bit, A lot, Very much
- **Lack of accessibility/quality of health and social care:**
  Not at all, Little, A bit, A lot, Very much
- **Do you have difficulty acquiring new knowledge and skills?**
  Not at all, Little, A bit, A lot, Very much
- **How easily can you relax and distract yourself?**
  Enjoy hobbies easily, Hard to do previous hobbies, No time outside work, Get tired quickly outside home, Constant boredom from fatigue
- **How many opportunities do you have for leisure activities?**
  Very much, A lot, A bit, Little, Not at all
- **Transport:**
  Seamless, Difficulty driving alone, Difficulty using public transport, Difficulty walking outside alone, No independent transport

Supplementary Material S3: Physical Stress Test

This test was developed by the principal investigator. Participants were asked to indicate their typical values or habits in the last month for the items listed below. Each item includes the following set of predefined response ranges:

- Daily driving time (min): *0–30, 30–60, 60–90, 90–120, 120+*
- Computer time for business (min): *0–60, 60–120, 120–180, 180–300, 300+*
- Computer time for social purposes (min): *0–60, 60–120, 120–180, 180–300, 300+*

- Walking for transportation (min): *0–20, 20–40, 40–60, 60–90, 90+*
- Walking for sports (at least 30 min per week): *0–1, 1–2, 2–3, 3–5, 7*
- Number of days of walking or running for more than one hour: *None, 1–2, 3–4, 5–6, 7*
- Walking for sports (mountain/hill): *None, 0–30, 30–60, 60–90, 90+*
- Does he or she do sports? *None, Aerobics at home, Yoga/Pilates, Gym, Professional*
- How many flights of stairs do you climb? *None, Elevator, 1, 2, 3+*
- Marital status: *Single, Engaged, Married, Divorced, Widow*
- Standing time at business place (min): *0–30, 30–60, 60–120, 120–180, 180+*
- Alcohol: *None, Once a week, 2–3 times a week, Everyday beer/wine, Excessive alcohol more than 3 times a week*
- How many kilos does he/she carry in shopping? *None, 0–5, 5–10, 10–15, 15+*
- How often does a housekeeper come? *Everyday, 2+ times a week, Once a week, Every 2 weeks, None*
- Number of children: *None, 1, 2, 3, 4+*
- Daily TV time (min): *None, 0–60, 60–180, 180–240, 240+*
- Daily working time while standing (min): *None, 0–60, 60–240, 240–360, 360+*
- Daily working time while sitting (desk job) (min): *None, 0–60, 60–240, 240–360, 360+*
- Years as a business owner: *None, 0–5, 5–10, 10–20, 20+*
- Years as an upper level manager: *None, 0–5, 5–10, 10–20, 20+*
- Financial income (Total household income): *50k+, 30–50k, 20–30k, 10–20k, below 10k*
- Smoking: *None, Sometimes, 0–10, 10–20, 20+*
- Daily sleep time (hr): *6–8, 8–10, 6–4, Less than 4, More than 10*
- Number of meals per day: *3, 2, 1, 4, 5+*
- Night work (hr): *None, 18–21, 21–24, 24–8, More than 3 times a week*

Supplementary Material S4: Disease Questionnaire

This test was also developed by the principal investigator. Participants were asked to indicate the status of the following conditions using one of the options: *No, Had in the past, Still have, Being treated, Surgery*.

- Hypertension
- Diabetes
- Cancer
- Obesity
- Depression
- COPD—Asthma
- Osteoporosis
- Paralysis
- Parkinson’s Disease
- Autoimmune Disease
- Thyroid Disease
- Pulmonary Disease
- Cardiac Disease
- Liver Disease
- Pancreas Disease
- Gastric Diseases
- Intestinal and Gall Bladder Disease
- Colon Disease
- Renal and Bladder Disease
- Genital Organ Disease
- Rheumatic Disease

- Childhood-onset Chronic Diseases
- Infectious Diseases (including HIV)
- Covid-19
- Cervical Disc Problems
- Back Problems
- Lumbar Disc Problems
- Shoulder Problems
- Elbow Problems
- Disorders of the Pelvis
- Knee Problems
- Ankle/Foot Problems
