# Supplementary figures and images for: The Effect of a Personalized Exercise Program on Muscle Functional Capacity and Quality of Daily Life: A Randomized Pilot Study
Source: Int J Environ Res Public Health. 2025 Aug 28;22(9):1344. doi: 10.3390/ijerph22091344 (PMC12469410; doi:10.3390/ijerph22091344)

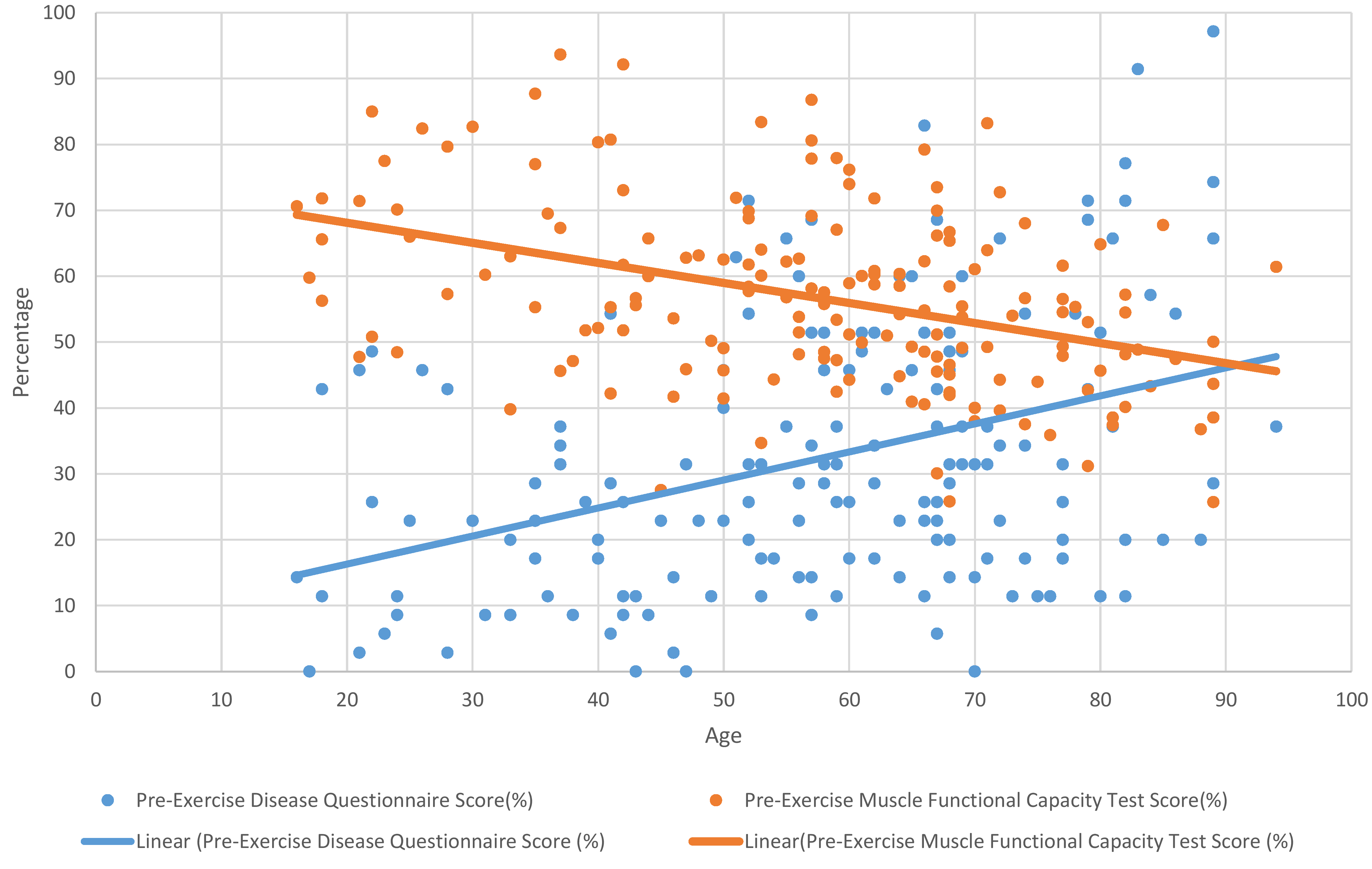

Supplement: Supplementary file 1 [file ijerph-22-01344-s001.zip › Definitions/Figure1.png]

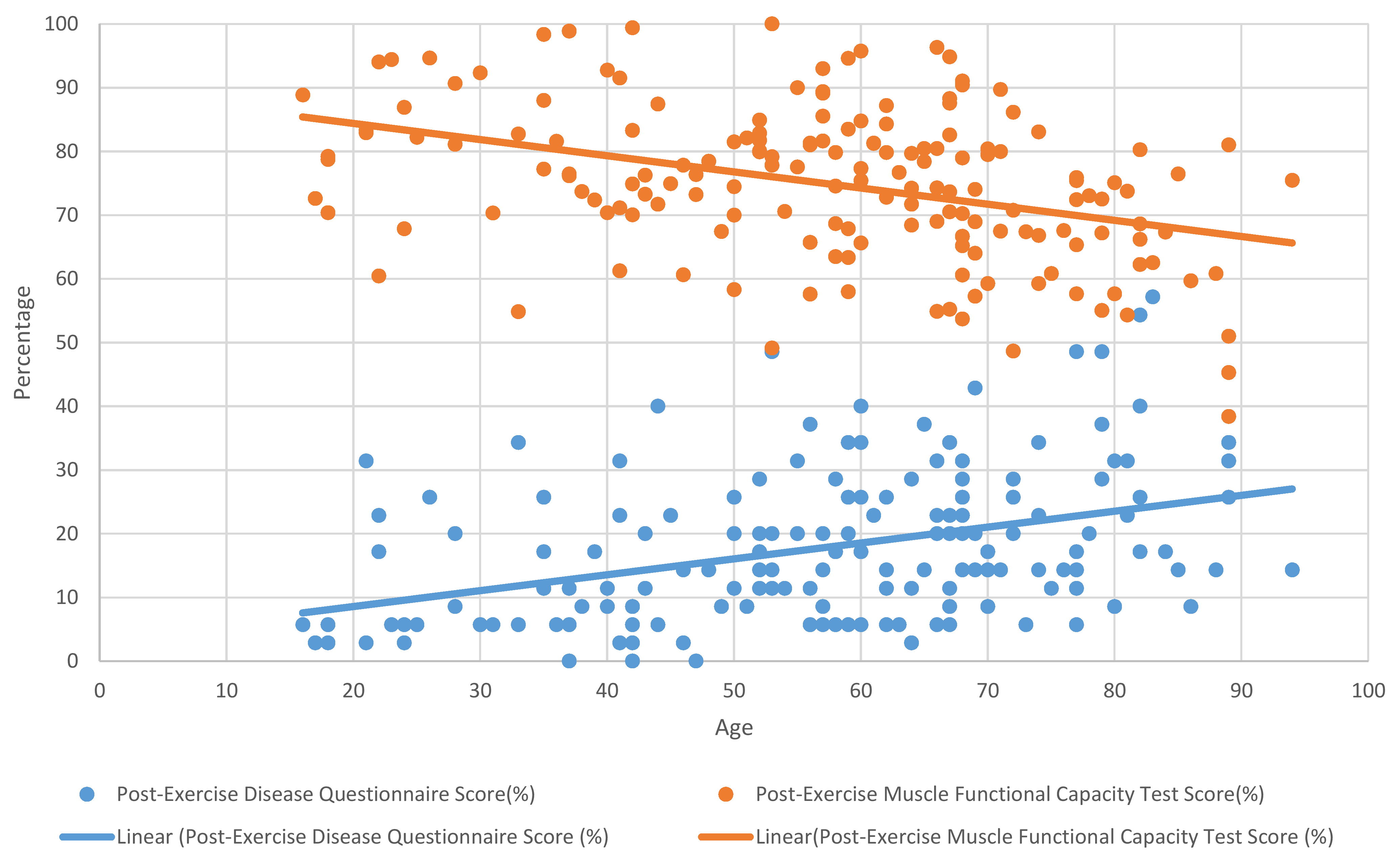

Supplement: Supplementary file 1 [file ijerph-22-01344-s001.zip › Definitions/Figure2.png]

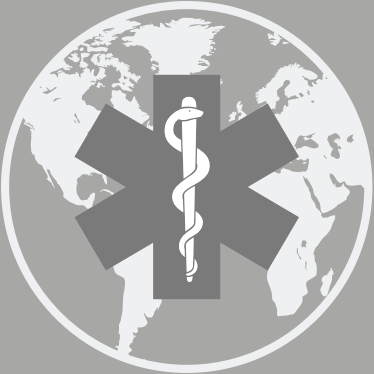

International Journal of  
*Environmental Research  
and Public Health*

Supplement: Supplementary file 1 [file ijerph-22-01344-s001.zip › Definitions/ijerph-logo-eps-converted-to.pdf]

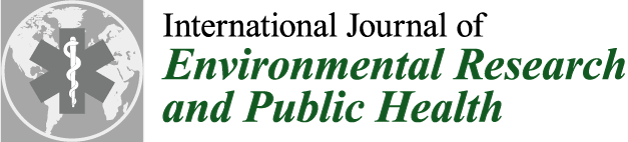

Supplement: Supplementary file 1 [file ijerph-22-01344-s001.zip › Definitions/ijerph-logo.png]

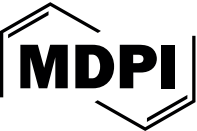

Supplement: Supplementary file 1 [file ijerph-22-01344-s001.zip › Definitions/logo-mdpi-eps-converted-to.pdf]

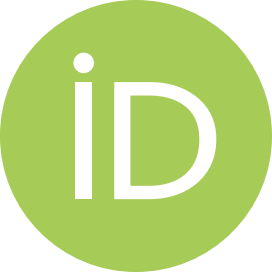

Supplement: Supplementary file 1 [file ijerph-22-01344-s001.zip › Definitions/logo-orcid.pdf]

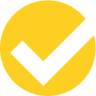

check for  
updates

Supplement: Supplementary file 1 [file ijerph-22-01344-s001.zip › Definitions/logo-updates-eps-converted-to.pdf]
